# Insights from electronic health record data to improve mental health service delivery during the COVID-19 pandemic

**DOI:** 10.1192/j.eurpsy.2021.131

**Published:** 2021-08-13

**Authors:** R. Patel, J. Irving, A. Brinn, M. Broadbent, H. Shetty, M. Pritchard, J. Downs, R. Stewart, R. Harland

**Affiliations:** 1 Department Of Psychosis Studies, Institute of Psychiatry, Psychology & Neuroscience, KCL, London, United Kingdom; 2 Nihr Maudsley Biomedical Research Centre, South London and Maudsley NHS Foundation Trust, London, United Kingdom; 3 Department Of Psychological Medicine, Institute of Psychiatry, Psychology & Neuroscience, KCL, London, United Kingdom

**Keywords:** telepsychiatry, telemedicine, SARS-Cov2, Electronic health records

## Abstract

**Background:**

Remote consultation technology has been rapidly adopted due to the COVID-19 pandemic. However, some healthcare settings have faced barriers in implementation. We present a study to investigate changes in rates of remote consultation during the pandemic using a large electronic health record (EHR) dataset.

**Methods:**

The Clinical Record Interactive Search tool (CRIS) was used to examine de-identified EHR data of people receiving mental healthcare in South London, UK. Data from around 37,500 patients were analysed for each week from 7^th^ January 2019 and 20^th^ September 2020 using linear regression and locally estimated scatterplot smoothing (LOESS) to investigate changes in the number of clinical contacts (in-person, remote or non-attended) with mental healthcare professionals and prescribing of antipsychotics and mood stabilisers. The data are presented in an interactive dashboard: http://rpatel.co.uk/TelepsychiatryDashboard.

**Results:**

The frequency of in-person contacts was substantially reduced following the onset of the pandemic (β coefficient: -5829.6 contacts, 95% CI -6919.5 to -4739.6, p<0.001), while the frequency of remote contacts increased significantly (β coefficient: 3338.5 contacts, 95% CI 3074.4 to 3602.7, p<0.001). Rates of remote consultation were lower in older adults than in working age adults, children and adolescents. Despite the increase in remote contact, antipsychotic and mood stabiliser prescribing remained at similar levels.

**Figure:**
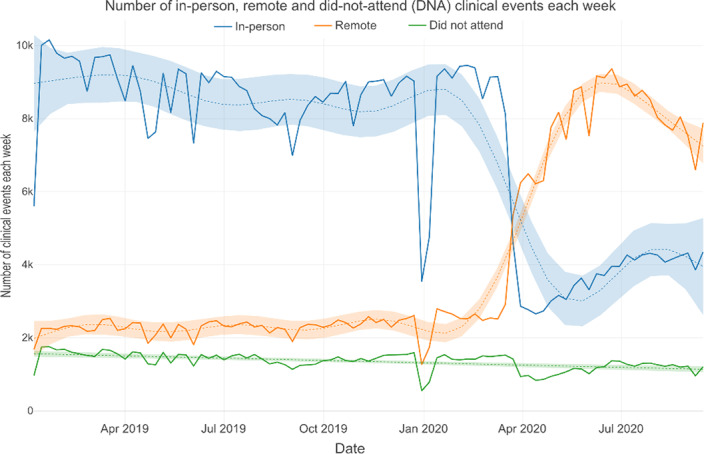

**Conclusions:**

The COVID-19 pandemic has been associated with a marked increase in remote consultation, particularly among younger patients. However, there was no evidence that this has led to changes in prescribing. Further work is needed to support older patients in accessing remote mental healthcare.

**Disclosure:**

All authors have completed the ICMJE uniform disclosure form at www.icmje.org/coi_disclosure.pdf and declare: RS has received funding from Janssen, GSK and Takeda outside the submitted work. RP has received funding from Janssen, Induction Healthcare and H

